# Transcranial direct current stimulation combined with cognitive-behavioral interventions for depressive disorders: a systematic review and meta-analysis of sham-controlled randomized trials

**DOI:** 10.3389/fpsyt.2026.1899231

**Published:** 2026-08-26

**Authors:** Otavio A. F. S. Da Silva, Samuel I. D. S. Pereira, Alper Mert, Débora R. de Aguiar, Mayara B. Nogueira, Gabriela A. Prilip, Marielia Barbosa L. de Freitas, Dennison C. Monteiro, Humberto M. M. Dos Santos, Gilberto S. Alves

**Affiliations:** 1 Translational Psychiatry Research Group, Universidade Federal do Maranhão - UFMA, São Luís, Brazil; 2 Medicine, University of Buenos Aires, Buenos Aires, Argentina; 3 Psychiatry, Nazilli State Hospital, Aydin, Türkiye; 4 Psychology, Universidade Federal de São Paulo - UNIFESP, São Paulo, Brazil; 5 Psychology, Centro Universitário São Camilo, São Paulo, Brazil; 6 Psychiatry, Faculty of Medical Sciences, University of Pernambuco - UPE, Recife, Brazil; 7 Psychiatry, University of Porto, Porto, Portugal

**Keywords:** cognitive behavioral therapy, cognitive control training, depressive disorders, interventions, transcranial direct current stimulation

## Abstract

**Introduction:**

Depressive disorders are a prevalent psychiatric condition; major depressive disorder (MDD) specifically affects approximately 5.7% of adults worldwide and is associated with high levels of disability and disease burden. Although effective interventions are available, some patients do not achieve adequate remission or response rates. In such cases, clinicians often combine interventions to enhance outcomes. This systematic review evaluated whether adding transcranial direct current stimulation (tDCS) to cognitive-behavioral interventions (CBIs) reduces depressive symptoms and improves response and remission rates versus sham tDCS in a sample of patients with depressive disorders.

**Methods:**

We searched ClinicalTrials.gov, Embase, Scopus, PubMed, Web of Science, Cochrane, PsycINFO, and Ovid from inception to October 2025. The primary outcome was reduction in depressive symptoms; response and remission rates were secondary outcomes. Standardized mean difference (SMD) and risk ratio (RR) were used as effect measures for continuous and dichotomous outcomes, respectively, within a random-effects model. Only randomized controlled trials (RCTs) selected according to the PICO framework were included.

**Results:**

Ten RCTs comprising 321 patients were included. Primary analysis showed no significance between intervention and control in a sample with MDD as primary diagnosis (SMD: −0.05; 95% CI: −0.44 to 0.34; I² = 26%). Secondary analysis did not demonstrate a significant advantage over the control group for any reported outcome: depressive symptoms (SMD = –0.03; 95% CI –0.30 to 0.23; I² = 6%), response (RR = 1.08; 95% CI 0.67 to 1.74; I² = 0%), or remission (RR = 1.33; 95% CI 0.74 to 2.38; I² = 0%). Subgroup analyses of cognitive behavioral therapy (CBT) and cognitive control training (CCT) also showed no significant advantage.

**Conclusions:**

In sham-controlled trials, current evidence does not demonstrate a reliable incremental benefit of adjunctive tDCS over sham when paired with heterogeneous CBIs in patients with depressive disorders. These findings should be interpreted with caution because of methodological heterogeneity in intervention parameters and insufficient statistical power.

**Systematic review registration:**

https://osf.io/bf4qt/overview, identifier, 10.17605/OSF.IO/BF4QT

## 1 Introduction

Depressive disorders are prevalent and debilitating psychiatric conditions; major depressive disorder (MDD) specifically affects approximately 5.7% of adults worldwide (1). It is associated with substantial personal suffering and economic burden (2). Diagnosis is based on clinical evaluation of symptom clusters, guided by the Diagnostic and Statistical Manual of Mental Disorders (DSM-5) and the International Classification of Diseases (ICD-11) (3, 4). Core symptoms of depressive episodes comprise persistent depressed mood, anhedonia, and disturbances in appetite, sleep, and cognition. Suicidal ideation and self-harming behaviors may occur across different levels of symptom severity, although they are generally associated with greater clinical burden and risk (3, 5).

Although depressive disorders have a multifactorial and incompletely understood etiology, several pathophysiological models have been proposed. These include the monoaminergic hypothesis, dysregulation of the hypothalamic–pituitary–adrenal (HPA) axis, neuroinflammation, genetic and epigenetic vulnerability, and structural and functional brain alterations (5–9). Depressive disorders also pose a significant public health concern due to their strong association with suicide, which is currently the third leading cause of death among individuals aged 15 to 29 years (1, 10). First-line treatments for depressive disorders include antidepressants (11) and psychological interventions, particularly cognitive and behavioral approaches such as cognitive behavioral therapy (CBT), cognitive control training (CCT), and behavioral activation (12). The combination of both has also been shown to be effective in preventing relapse (13, 14).

Neuromodulation techniques, including transcranial direct current stimulation (tDCS) and transcranial magnetic stimulation (TMS), are also used in depressive disorders and have shown promising results (15–17), especially when combined with existing treatments (18–20). tDCS is a non-invasive neuromodulation technique in which a low-intensity electrical current is applied to the scalp to modulate cortical excitability in specific brain regions (21, 22). It has been used for several neurological and psychiatric conditions, including Parkinson’s disease and schizophrenia (23), and can be applied alone or as an adjunct to established treatments such as antidepressants or psychotherapy (21, 24).

Despite available treatments, many patients do not achieve a response or remission, highlighting the need for additional options (25, 26). In this context, augmentation — defined as combining interventions with usual care to enhance their effects — emerges as a potential strategy (27). Neuromodulation may enhance the cognitive processes targeted by psychotherapy, suggesting potential synergy (20, 28). While tDCS is generally safe, well-tolerated, low-cost, and portable, especially compared to other neuromodulation techniques such as electroconvulsive therapy or TMS, its efficacy as an adjunct to psychological interventions remains to be established (29, 30).

Several broader syntheses have evaluated tDCS combined with psychotherapy, pharmacotherapy, and psychosocial interventions (27, 31). However, it remains unclear whether active tDCS is superior to sham when paired with cognitive-behavioral interventions (CBIs) in depressive disorders, particularly given heterogeneity in setting, stimulation timing, and patient population. Given the neurobiological effects of tDCS and the cognitive targets shared across different types of CBIs, such as dysfunctional thought patterns in CBT and executive functions like attention and working memory in cognitive training, this review aimed to explore the viability of augmenting cognitive- and behavioral-based interventions with tDCS as a broad treatment concept for reducing depressive symptoms in a broader sample of patients with depressive disorders.

## 2 Methods

### 2.1 Study design

This systematic review and meta-analysis were conducted in accordance with the Cochrane Handbook for Systematic Reviews of Interventions and reported in accordance with the Preferred Reporting Items for Systematic Reviews and Meta-Analyses (PRISMA) guidelines. The review protocol was prospectively registered in the Open Science Framework (DOI: 10.17605/OSF.IO/BF4QT) and is fully accessible via the reference list (32).

### 2.2 Search strategy

The following eight databases were searched for records published from inception up to October 2025: PubMed, Embase, Cochrane Library, Web of Science, Scopus, ClinicalTrials.gov, Ovid, and PsycINFO. Searches were conducted using the following primary keywords: “Depressive Disorders”, “Transcranial Direct Current Stimulation”, “Cognitive”, “Behavioral” and “Interventions”. The complete search strategy and the list of included studies are provided in **Supplementary Tables 1** and **2**, respectively.

### 2.3 Eligibility criteria

We included only randomized controlled trials (RCTs) that investigated the use of tDCS augmented with CBIs in patients with depressive disorders. The eligibility criteria were based on the PICO framework: (P) Population: individuals with depressive disorders, including MDD and other depressive presentations with or without psychiatric comorbidities; (I) Intervention: active tDCS combined with CBIs; (C) Control: sham tDCS combined with CBIs; (O) Outcomes: reduction in depressive symptoms, assessed using validated depression rating scales such as the Montgomery–Åsberg Depression Rating Scale (MADRS) and the Beck Depression Inventory (BDI); response and remission rates; and improvements in patients’ quality of life (QOL), assessed using validated measures such as the Satisfaction with Life Scale (SWLS). Studies were included only if at least one of these outcomes was reported and if they were published in English. We excluded studies involving bipolar depression, non-randomized study designs, psychological interventions that were not cognitive or behavioral in nature, and studies that did not report any of the prespecified outcomes.

### 2.4 Study selection and data extraction

All title and abstract screening and data extraction were conducted independently by two reviewers (O.A. and S.I.) using a predefined search strategy, and any disagreements were resolved by consensus. Interrater agreement was assessed using Cohen’s kappa (κ = 0.81), indicating almost perfect agreement. WebPlotDigitizer was used to extract data from figures and graphs when the data were not directly available. When multiple eligible depression measures or time points were reported within a study, we selected the BDI score assessed at the end of the acute treatment period. This prioritization of BDI/BDI-II over other depression scales was not prespecified in the registered protocol and was decided *post hoc* based on data availability and consistency across included studies.

### 2.5 Statistical analysis

Statistical analyses were performed using Review Manager version 5.1.7 (Cochrane Collaboration). Standardized mean differences (SMDs) were used as the effect measure for all continuous outcomes, while risk ratios (RRs) were used for dichotomous outcomes, both with 95% confidence intervals. Random-effects models were applied throughout. Statistical heterogeneity was assessed using the Cochran Q test and the I² statistic, with p-values < 0.10 and I² > 25% considered indicative of significant heterogeneity.

The following analytical procedures were applied in accordance with Cochrane Handbook recommendations (33). For multi-arm trials in which comparisons against a shared control group were required, the control group was split into independent subgroups, preserving the original means and standard deviations. For dichotomous outcomes with zero-event cells, a continuity correction of 0.5 was applied to all cells of the corresponding 2×2 table. For studies reporting only baseline and post-intervention means and standard deviations, change-score variances were calculated assuming a correlation coefficient of 0.5 between pre and post intervention scores, as recommended when this value is not reported. Given the limited number of studies within each subgroup, the Hartung-Knapp-Sidik-Jonkman variance correction was applied to all random-effects models.

### 2.6 Risk of bias and certainty of evidence

Risk of bias was assessed using version 2 of the Cochrane Risk of Bias tool, and the certainty of evidence for primary outcomes was evaluated using the Grading of Recommendations, Assessment, Development and Evaluation (GRADE) approach with GRADEpro GDT. Both assessments were conducted independently by two reviewers (O.A and M.B), with disagreements resolved by consensus. Publication bias was intended to be assessed through funnel plot analyses; however, no outcome had a sufficient number of studies to permit a meaningful visual inspection of asymmetry.

## 3 Results

### 3.1 Study selection and characteristics

**Figure 1:**
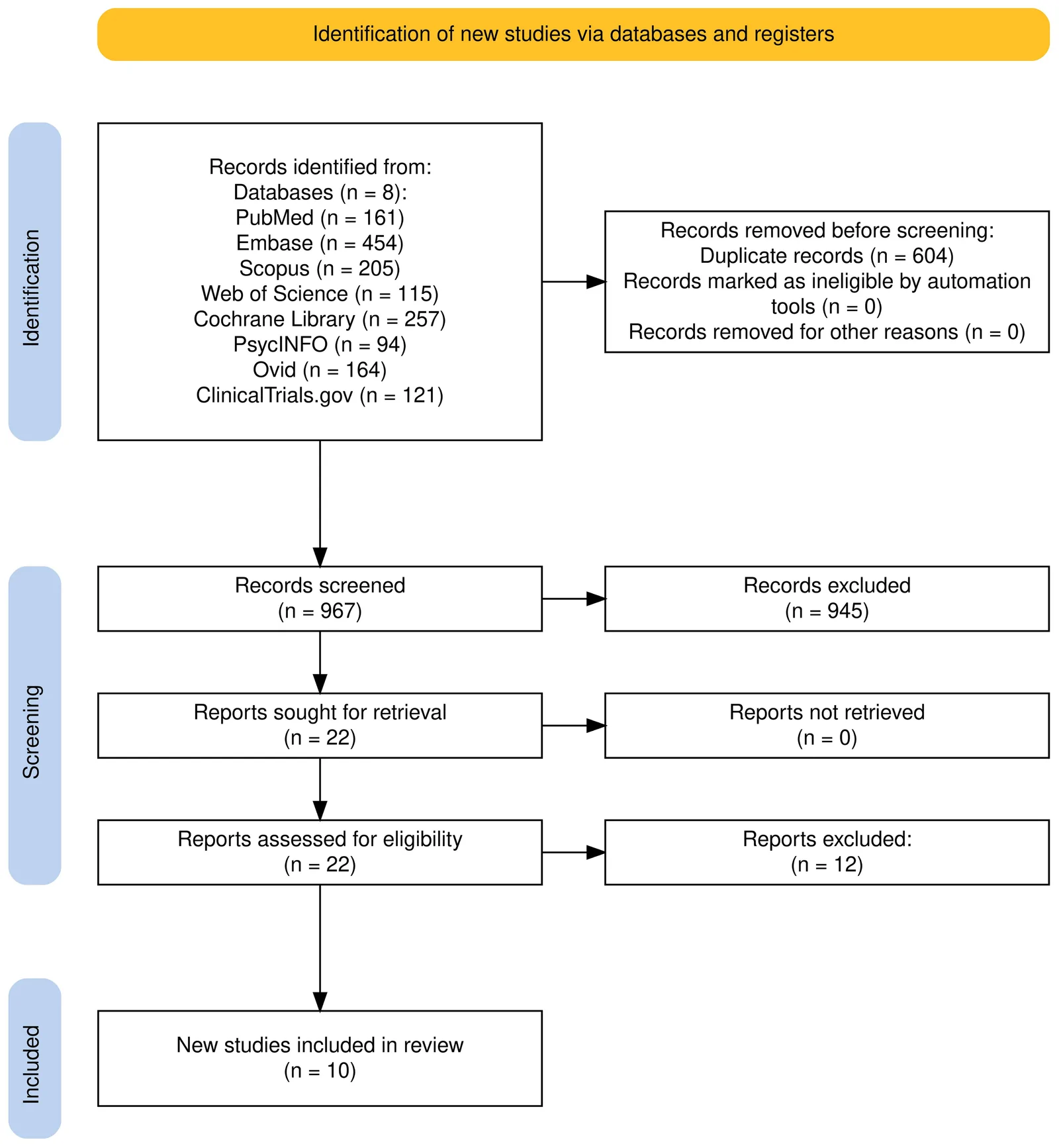
PRISMA flow diagram of study screening and selection.

Of 1,571 identified records, 604 duplicates were removed, leaving 967 for screening. Of the screened records, 22 underwent full-text review, and 12 were excluded for not meeting the inclusion criteria. Finally, ten RCTs (19, 34–42) were included in this systematic review and meta-analysis. Screening was conducted by assessing titles and abstracts using Rayyan software. **Figure 1** presents the PRISMA flow diagram.

A total of 321 patients were included: 168 received active tDCS and 153 received sham tDCS. **Table 1** summarizes the main characteristics of the 10 included studies, published between 2014 and 2025. Seven studies reported MDD as the primary diagnosis, whereas two reported acute depressive disorder and late-life depression. Among reported comorbidities, only anxiety disorders, such as specific phobia (SP) and agoraphobia (AGP), were identified. Cognitive decline was reported in only one study. The most applied stimulation intensity was 2 mA over the F3 and F4 scalp regions, corresponding to the left and right dorsolateral prefrontal cortices (DLPFC). CBT formats varied across studies, including individual, group-based, and internet-based delivery, while the Paced Auditory Serial Addition Test (PASAT) was the most common cognitive training intervention.

**Table 1:** Baseline characteristics of included trials.

Study	N (act/sham)	tDCS settings: current/N° sessions/duration/montage	C.I parameters: type/N° sessions/duration	Intervention	Control	Comorbidities	Primary diagnosis
Aust 2022 (34)	95 (48/47)	2mA/12/30min/F3 and F4	Group CBT/12/100min	tDCS + CBT	Sham tDCS + CBT	AGP, SP, HPC, SPD	MDD
Brunoni 2014 (35)	37 (20/17)	2mA/10/30min/F3 and F4	CCT (Modified PASAT)/10/15min	tDCS + CCT	Sham tDCS + CCT	Anxiety Disorders	Acute Depressive Disorder
Carvalho 2025 (19)	10 (6/4)	2mA/18/30min/F3 and F4	Individual CBT/12/60min	tDCS + CBT	Sham tDCS + CBT	None	MDD
Ha 2024 (41)	24 (12/12)	2mA/10/23min/F3	Workbook-based CT/10/NR	tDCS + CCT	Sham tDCS + CCT	Cognitive Decline	Elderly Depression
Nord 2019 (40)	39 (20/19)	1mA/8/20min/F3 and Ipsilateral Deltoid	Individual CBT/8/60min	tDCS + CBT	Sham tDCS + CBT	NR	MDD
Sararudi 2025 (36)	25 (13/12)	2mA/10/20min/F3 and mPFC	Individual CBT/12–20 sessions/45–90 min	tDCS + CBT	Sham tDCS + CBT	NR	SAD and Depression
Segrave 2014 (37)	18 (9/9)	2mA/5/24min/F3 and F8	CCT (Modified PSAT+Modified WAT)/5/24min	tDCS + CCT	Sham tDCS + CCT	NR	MDD
Sommer 2021 (42)	51 (17/17/17)	2mA and 1mA (2 arms)/12/23min/F3 and Ipsilateral Deltoid	CCT (Modified PASAT)/12/23min	tDCS + CCT	Sham tDCS + CCT	NR	MDD
Vanderhasselt 2015 (38)	33 (19/14)	2mA/10/30min/F3 and F4	NT (PASAT Variant)/10/15min	tDCS + CCT	Sham tDCS + CCT	Anxiety Disorders	MDD
Welch 2017 (39)	14 (9/5)	2mA/12/30min/F3 and F4	Individual eCBT/12/30min	tDCS + CBT	Sham tDCS + CBT	NR	MDD

AGP, agoraphobia; SP, specific phobia; HPC; hypochondria; SPD, somatoform pain disorder; NR, not reported; eCBT, computerized CBT; NT, neurocognitive training; CT, cognitive training; WAT, wells attentional training; PSAT, paced serial addition task and mA, milliampere.

### 3.2 Pooled analysis

**Figure 2:**
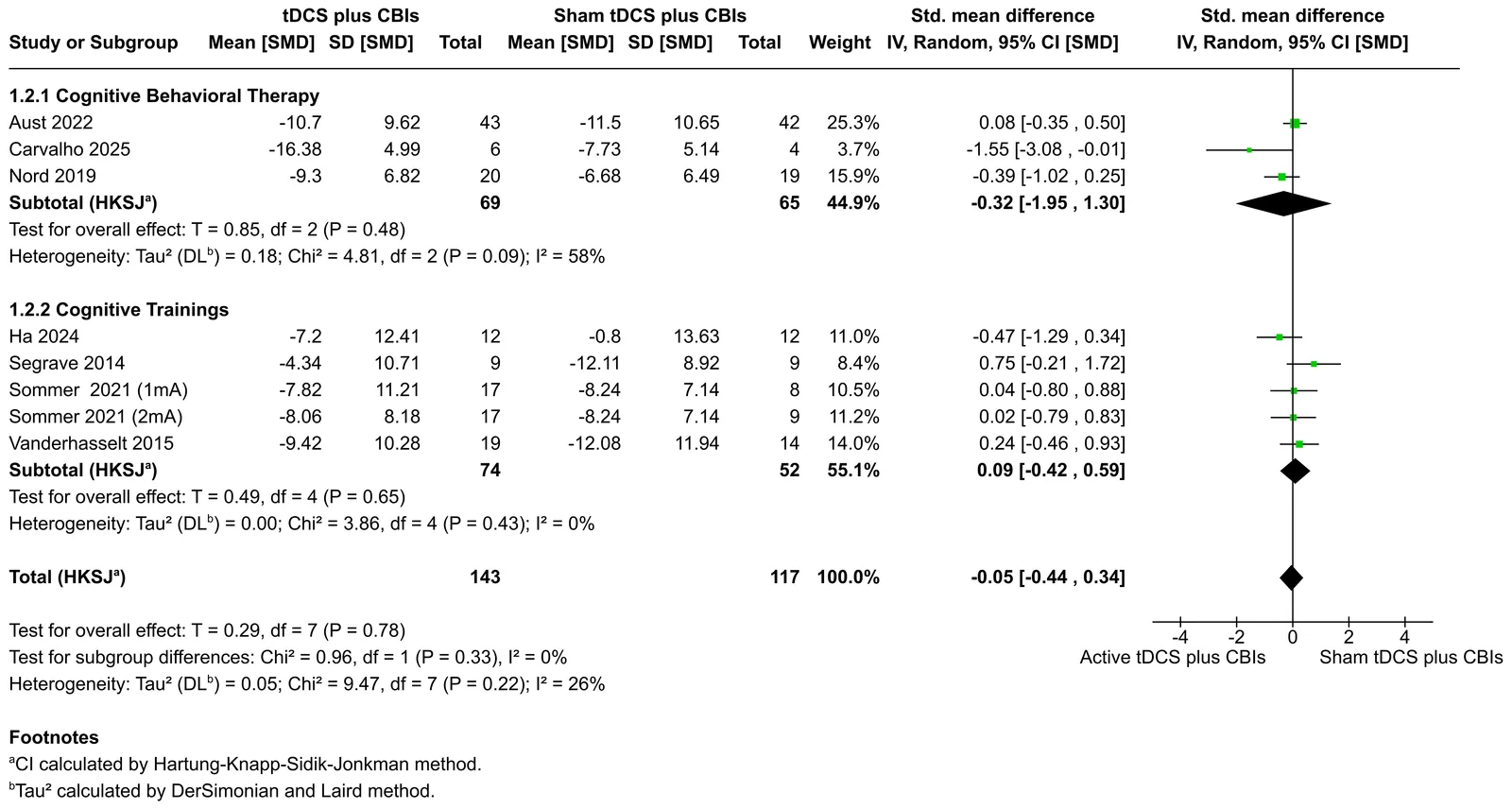
Forest plot of the primary analysis restricted to studies reporting MDD as the primary diagnosis, assessing the reduction in depressive symptoms between the adjunctive active tDCS and adjunctive sham tDCS groups.

Pooled analysis showed no significant difference between active and sham tDCS combined with CBIs in reducing depressive symptoms among patients with MDD as the primary diagnosis (SMD: −0.05; 95% CI: −0.44 to 0.34; I^2^ = 26%). Subgroup analyses also showed no statistically significant differences for the CBT (SMD: −0.32; 95% CI: −1.95 to 1.30; I^2^ = 58%) or CCT (SMD: 0.09; 95% CI: −0.42 to 0.59; I^2^ = 0%) groups. The corresponding forest plot is presented in **Figure 2**.

**Figure 3:**
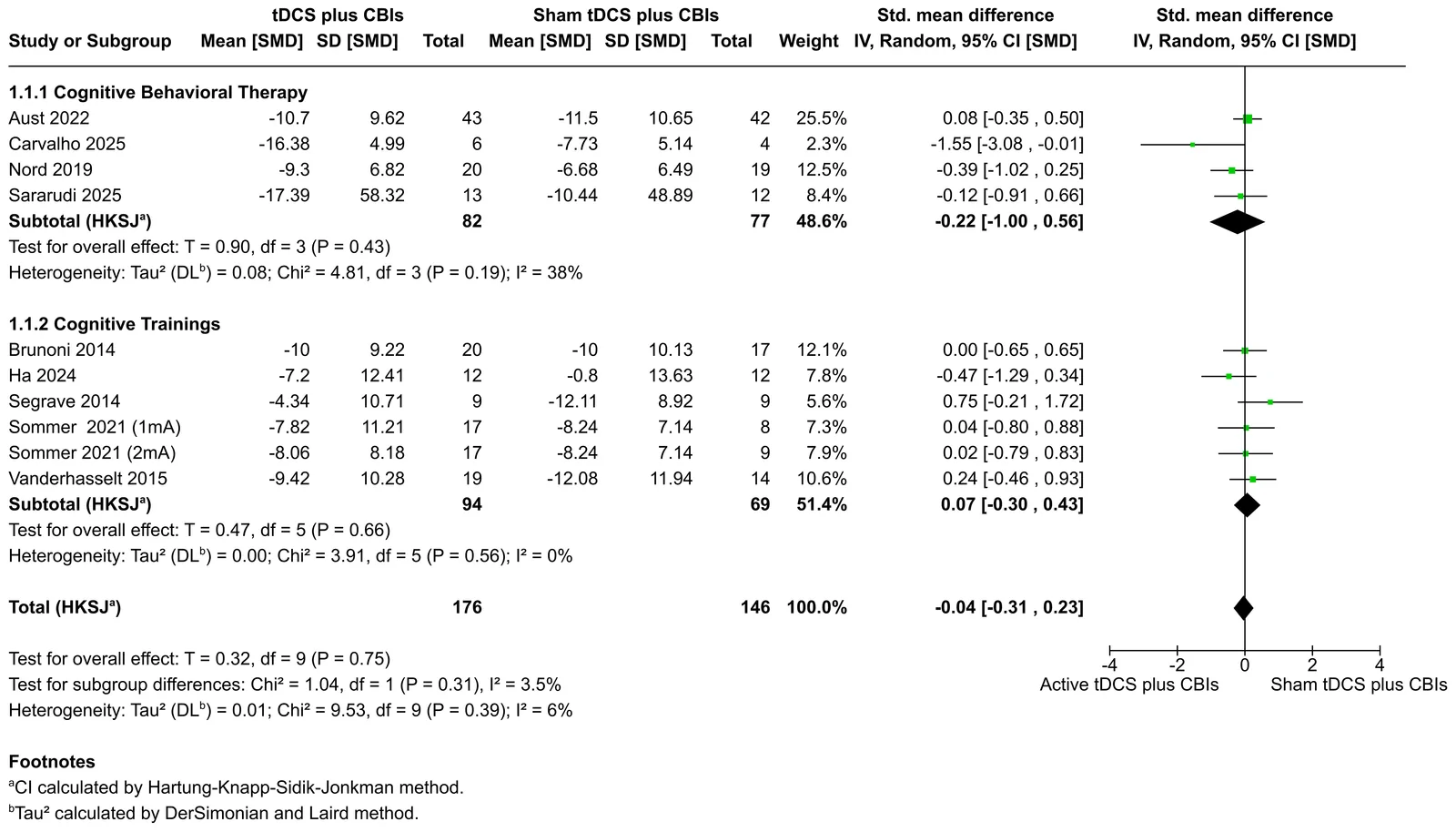
No statistically significant difference in depressive symptom reduction was observed between the adjunctive active tDCS and adjunctive sham tDCS groups in patients with depressive disorders.

**Figure 4:**
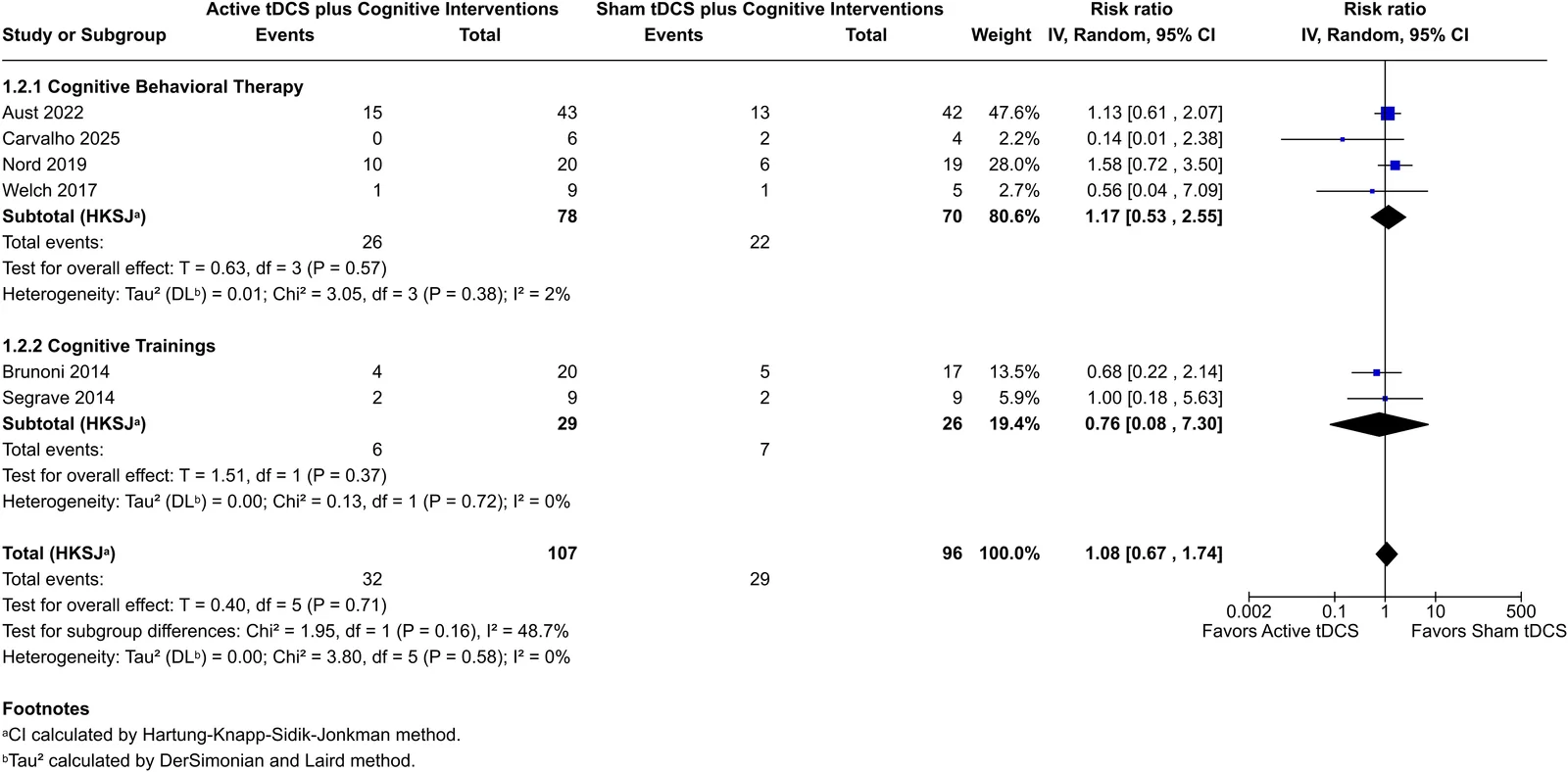
Response rates did not differ significantly between the adjunctive active tDCS and adjunctive sham tDCS groups in patients with depressive disorders.

**Figure 5:**
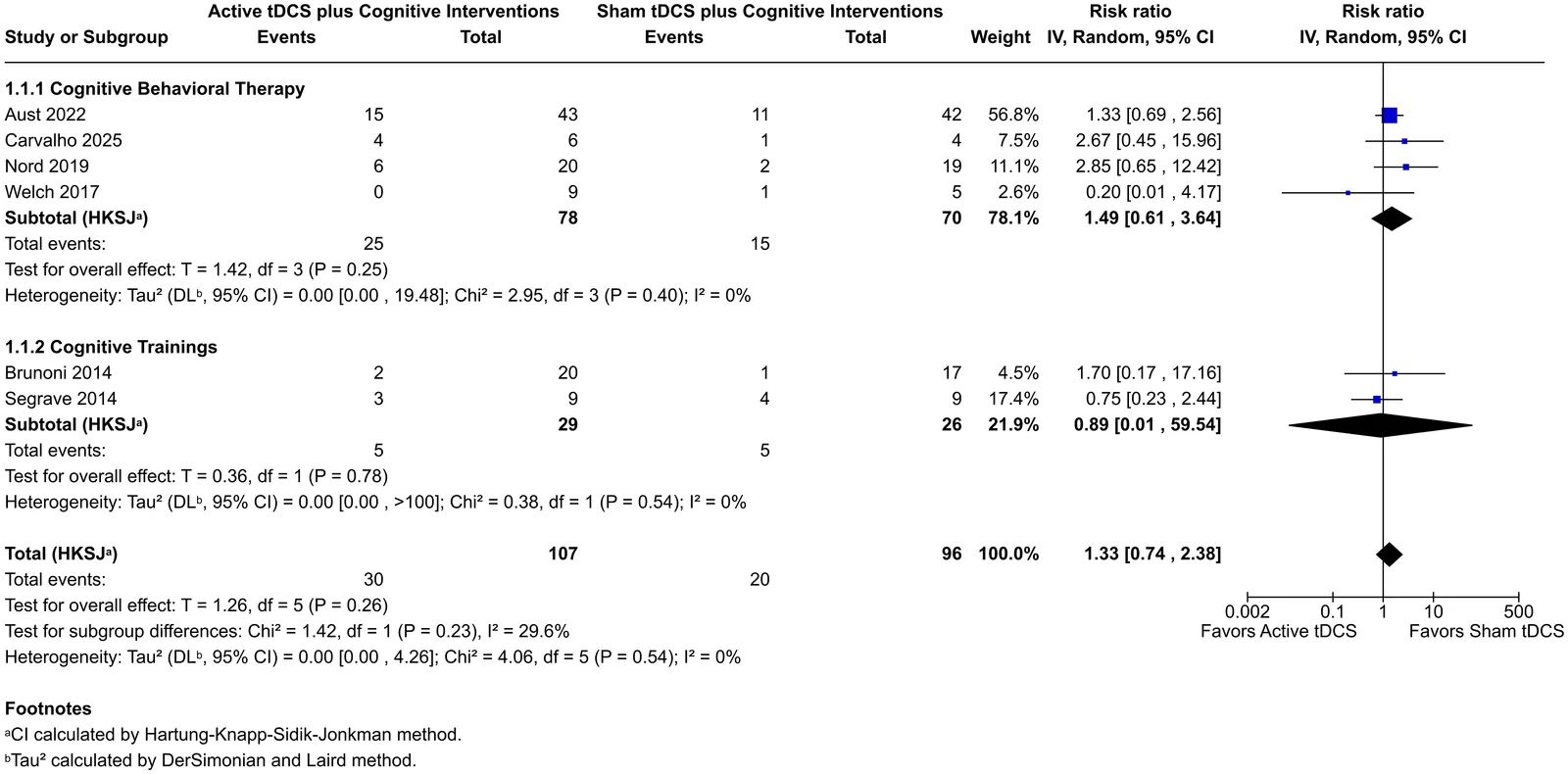
Remission rates did not differ significantly between the adjunctive active tDCS and adjunctive sham tDCS groups in patients with depressive disorders.

**Figure 6:**
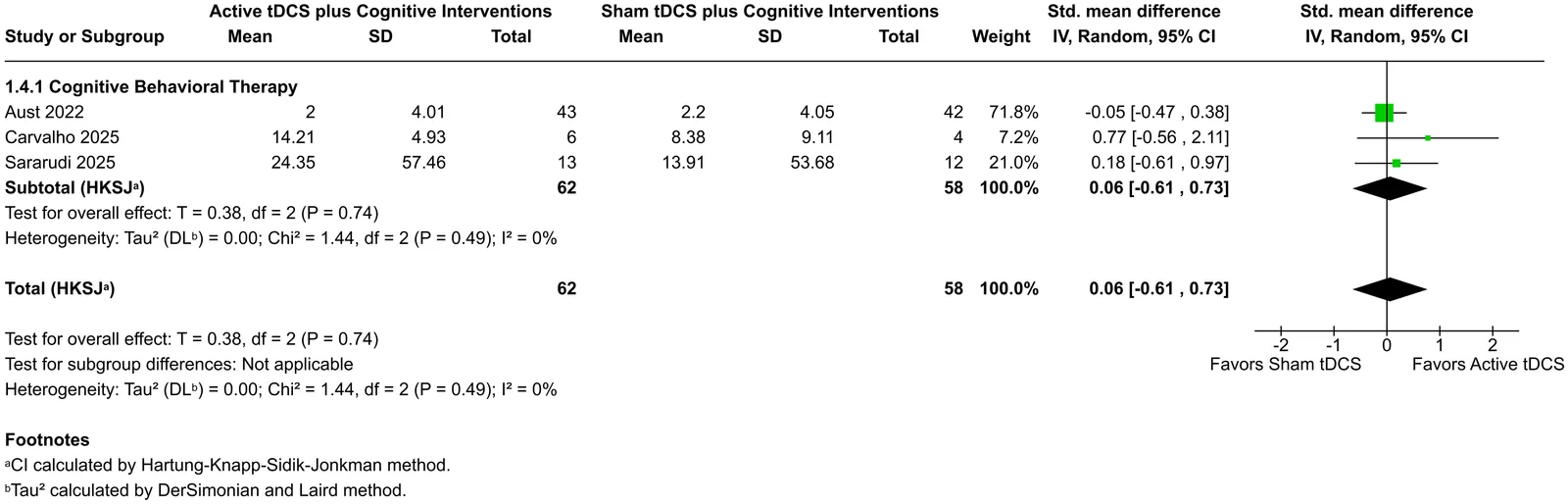
Quality of life did not differ significantly between the adjunctive active tDCS and adjunctive sham tDCS groups in patients with depressive disorders.

Also, secondary analysis showed, in the broader sample encompassing all depressive disorders presentations, no significant difference between active and sham tDCS combined with CBIs in reducing depressive symptoms (SMD: −0.04; 95% CI: −0.31 to 0.23; I^2^ = 6%) (**Figure 3**). The intervention group also showed no benefit over the control group for response (RR: 1.08; 95% CI: 0.67 to 1.74; I^2^ = 0%) (**Figure 4**), remission (RR: 1.33; 95% CI: 0.74 to 2.38; I^2^ = 0%) (**Figure 5**), or QOL (SMD: 0.06; 95% CI: −0.61 to 0.73; I^2^ = 0%) (**Figure 6**). In the CBT subgroup, no significant between-group differences were observed for depressive symptoms (SMD: −0.22; 95% CI: −1.00 to 0.56; I^2^ = 38%) (**Figure 3**), response (RR: 1.17; 95% CI: 0.53 to 2.55; I^2^ = 2%) (**Figure 4**), or remission (RR: 1.49; 95% CI: 0.61 to 3.64; I^2^ = 0%) (**Figure 5**). Similarly, the CCT subgroup showed no significant between-group differences in depressive symptoms (SMD: 0.07; 95% CI: −0.30 to 0.43; I^2^ = 0%) (**Figure 3**), response (RR: 0.76; 95% CI: 0.08 to 7.30; I^2^ = 0%) (**Figure 4**), or remission (RR: 0.89; 95% CI: 0.01 to 59.54; I^2^ = 0%) (**Figure 5**). Confidence intervals for remission in the CCT subgroup were wide, likely reflecting the small number of studies and patients.

### 3.3 Risk of bias and quality assessment

**Figure 7:**
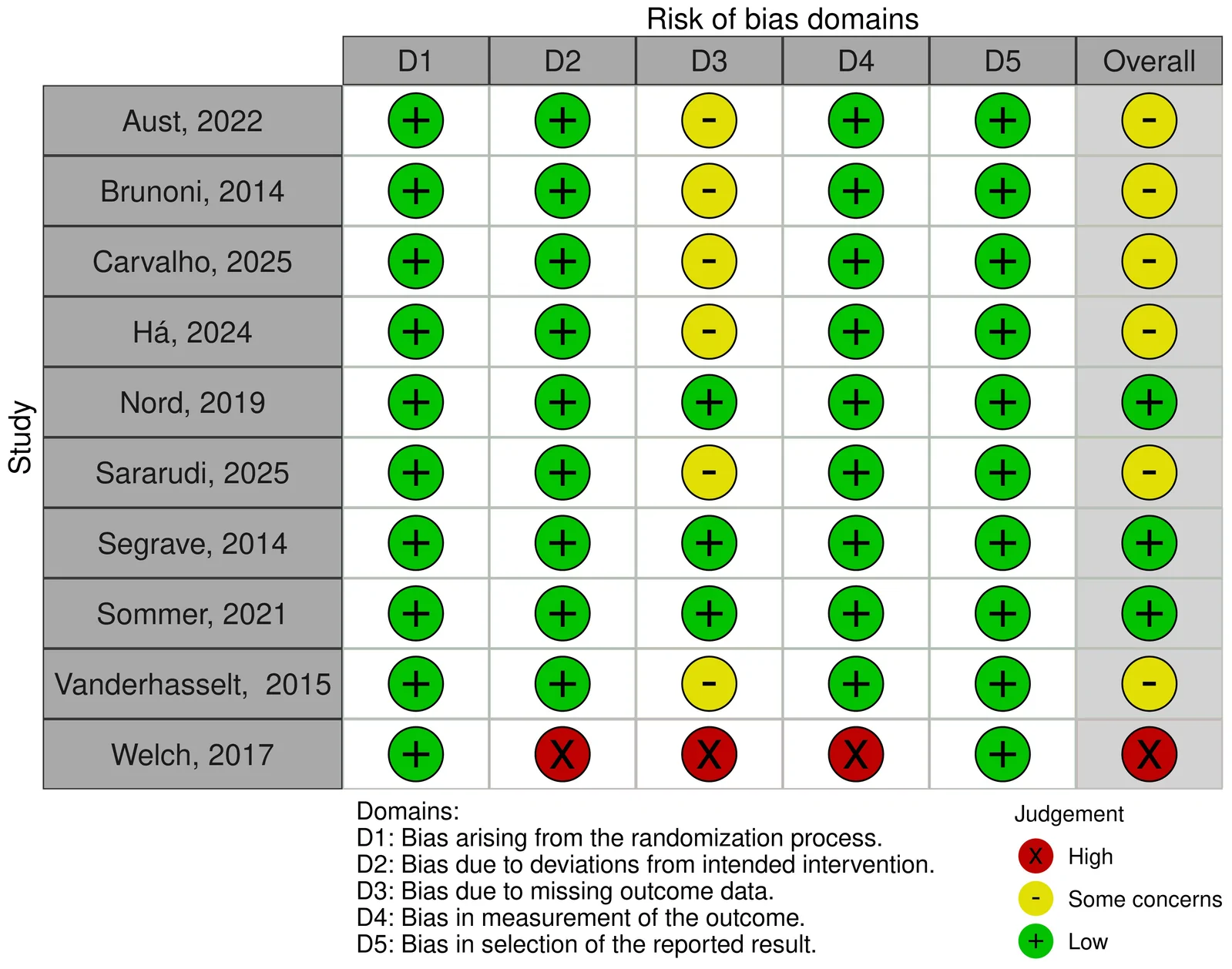
The risk of bias framework of all included studies.

The risk of bias assessment showed that six studies (19, 34–36, 38, 41) reported some concerns in the missing outcome data domain, while one study reported a high risk in the same domain (39), and three studies were rated as having a low risk of bias (37, 40, 42). The certainty of the evidence was assessed using the GRADE approach, with results indicating low to very low certainty. A summary of the risk of bias assessment is presented in **Figure 7**, and a summary of the GRADE assessment is presented in **Supplementary Table 5**.

### 3.4 Sensitivity analysis

**Figure 8:**
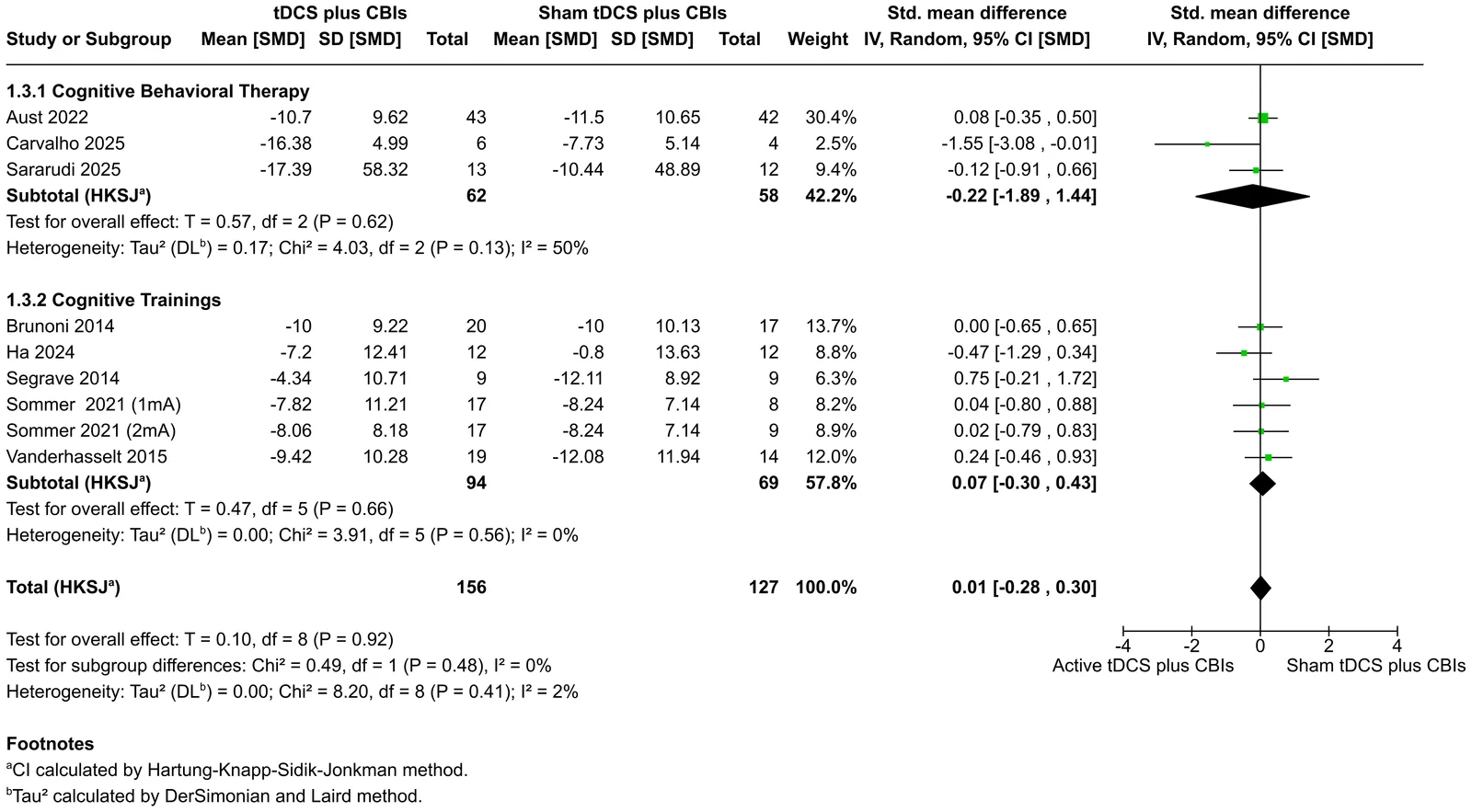
Sensitivity analysis including only studies that used the Beck Depression Inventory (BDI) as the depressive symptom outcome measure, assessing the outcome of reduction in depressive symptoms.

**Figure 9:**
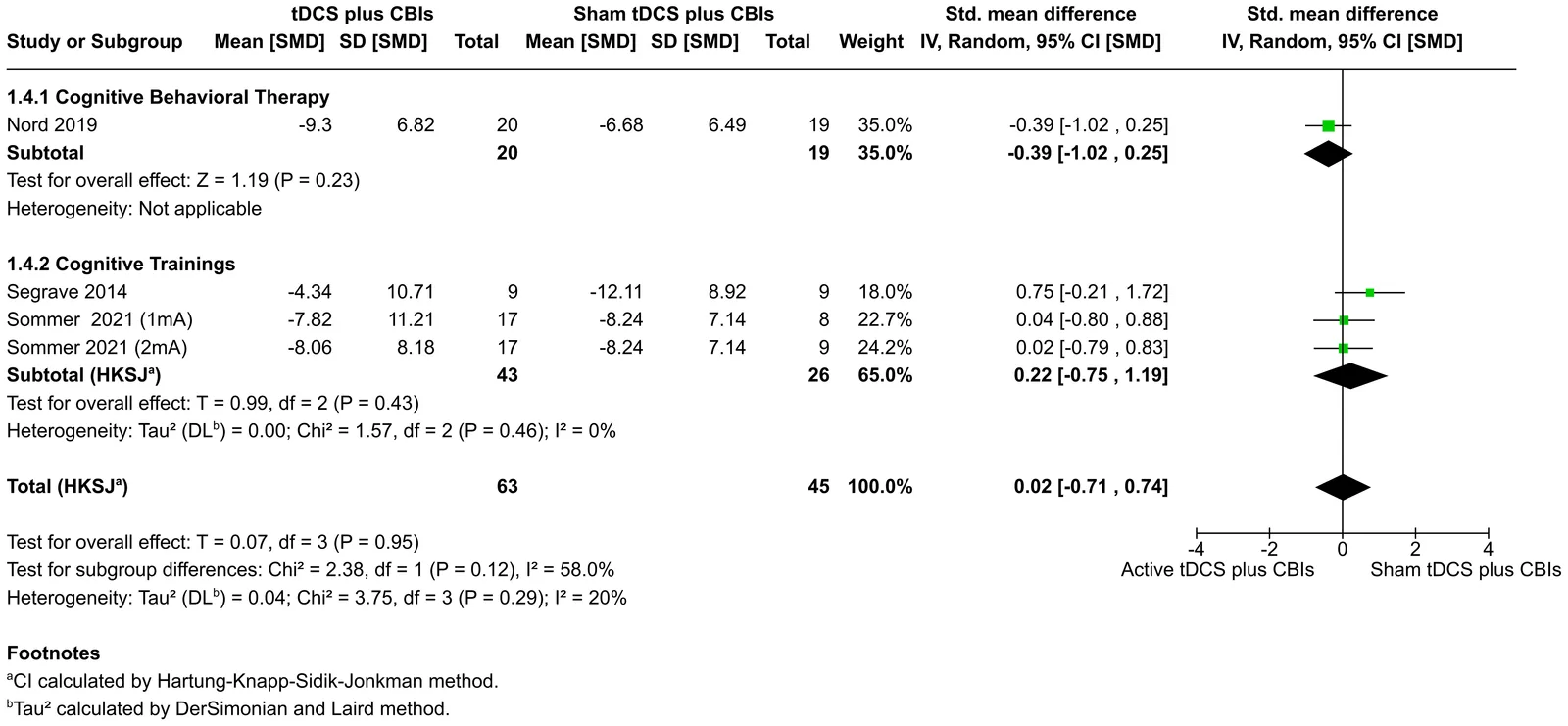
Sensitivity analysis including only studies classified as having a low risk of bias according to RoB 2, assessing the outcome of reduction in depressive symptoms.

**Figure 10:**
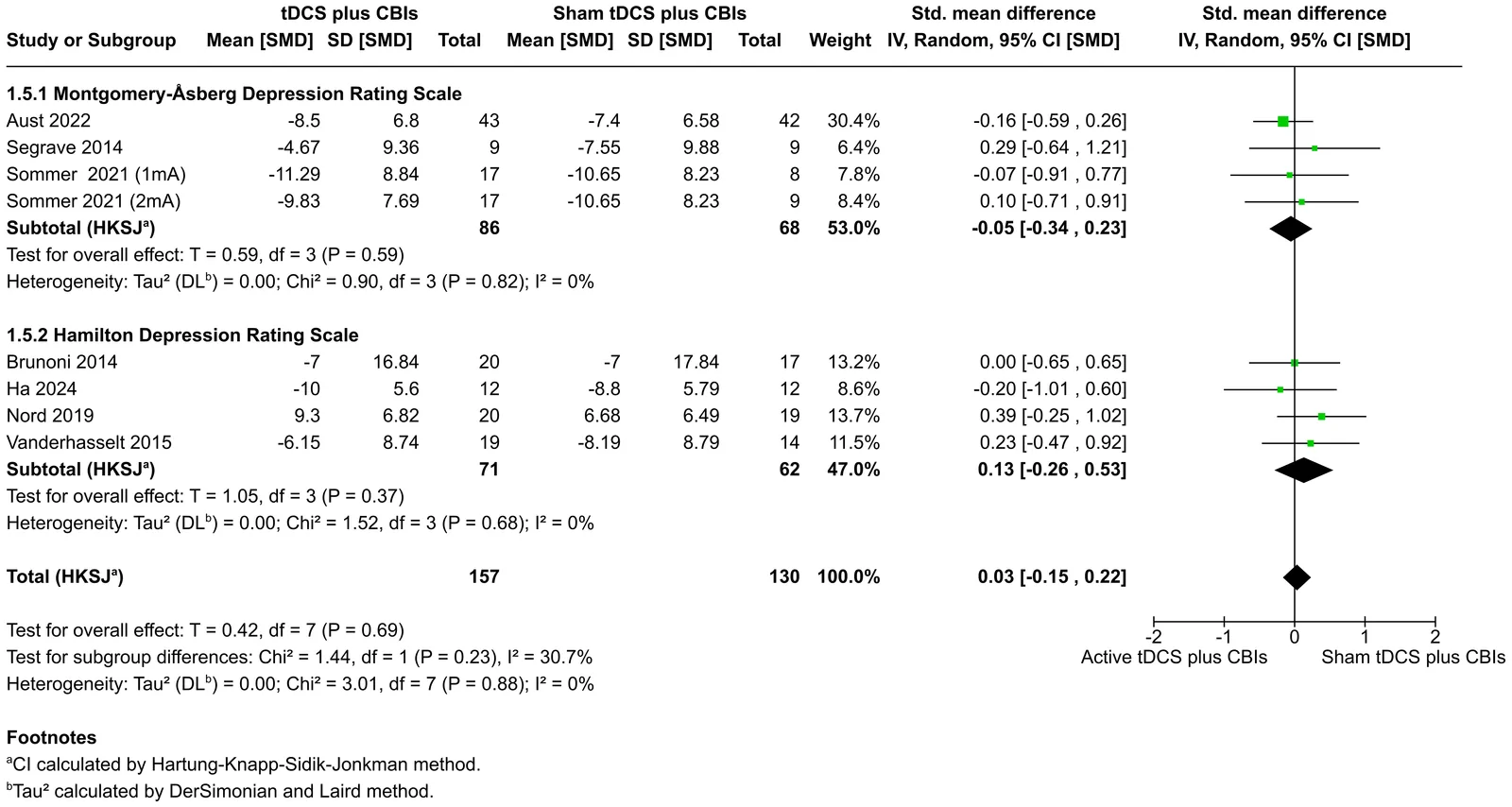
Sensitivity analysis restricted to studies using clinician-rated scales, such as the MADRS and HAM-D, as the depressive symptom outcome measure, assessing the reduction in depressive symptoms.

**Figure 11:**
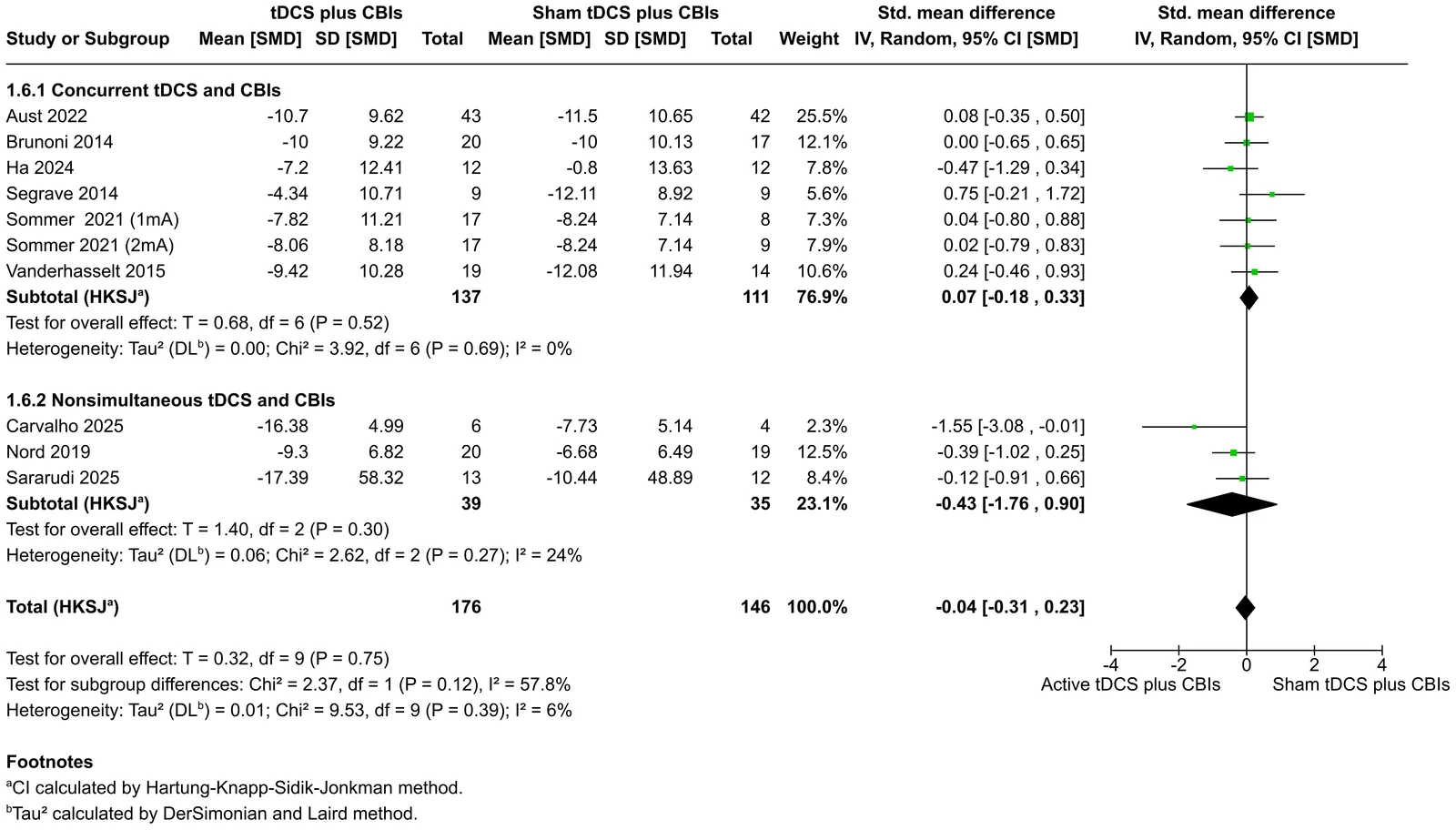
Sensitivity analysis comparing studies according to the timing of intervention delivery, with tDCS administered either concurrently with CBIs or separately, assessing the reduction in depressive symptoms.

**Figure 12:**
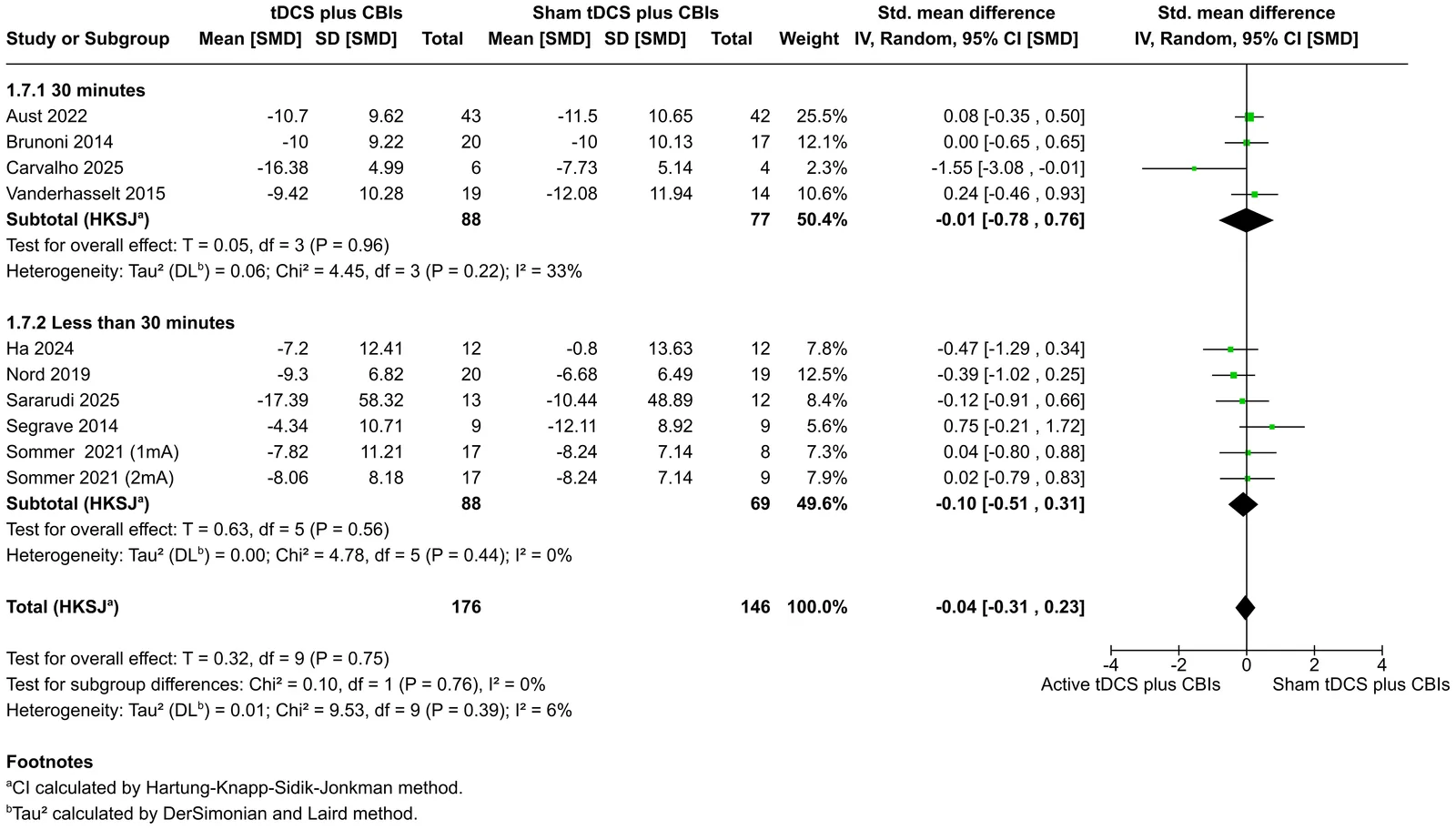
Sensitivity analysis comparing studies according to tDCS session duration, assessing the reduction in depressive symptoms.

Sensitivity analysis for the outcome of reduction in depressive symptoms was conducted by restricting the sample to studies reporting the BDI or BDI-II scale, and the results remained non-significant (**Figure 8**). Additionally, sensitivity analyses were conducted restricting the analysis to studies classified as having a low risk of bias (**Figure 9**), studies using clinician-rated scales (**Figure 10**), the timing of intervention delivery (**Figure 11**), and tDCS session duration (**Figure 12**). As in the previous analyses, no between-group differences were observed.

## 4 Discussion

### 4.1 Summary of main findings

In this systematic review and meta-analysis, we evaluated whether combining tDCS with CBIs reduces depressive symptoms in patients with depressive disorders. The findings indicate that combining tDCS with CBIs is no more effective than sham tDCS in reducing depressive symptoms or improving response and remission rates. Subgroup analyses were also conducted for patients receiving CBT or CCT, and no significant mean differences were observed between groups. To further explore the heterogeneity related to this augmentation strategy, including intervention format and parameters such as timing of application and outcome scale type, we conducted additional sensitivity analyses, none of which showed statistically significant differences between groups. This review suggests that the combination may not confer additional clinical benefit over sham stimulation.

Although tDCS, CBT, and CCT may each be effective when used alone, evidence does not show significant benefits for their combination (43–50). Consistent with our findings, previous meta-analyses also reported that this combination may be safe but not effective in patients with MDD (27, 31). Similarly, combining other brain stimulation modalities, such as repetitive TMS, with non-pharmacological interventions has also failed to demonstrate additional benefits (51, 52).

By contrast, one meta-analysis suggests that acutely augmenting psychological interventions with medication or brain stimulation may produce small-to-moderate effects in transdiagnostic samples (53). Notably, subgroup analyses of somatic augmentation, including non-invasive brain stimulation, exercise, and controlled breathing, showed larger effect sizes. Additionally, the combination of tDCS or TMS with pharmacotherapy has demonstrated small-to-moderate effects (31, 53, 54).

Protocol variability may explain the lack of significant effects. Stimulation parameters, scalp targets, and psychotherapy format may influence the efficacy of this augmentation strategy (55). Previous evidence has suggested that heterogeneity in stimulation parameters, including electrode size and placement (e.g., F3 and F4, corresponding to the dorsolateral prefrontal cortex), may be associated with poorer depressive outcomes (31). Furthermore, sham tDCS has been associated with meaningful placebo effects in depressive disorders, which may attenuate between-group differences (55).

Application protocols varied considerably across studies, including concurrent administration of tDCS with psychological therapy (34, 35, 37–39, 41) or delivery at separate time points (19, 36, 40). The timing of tDCS application and parameters such as amperage and device type also varied widely across studies (19, 34–42). Psychotherapy and neurocognitive training formats also varied substantially, ranging from computerized CBT (39) to individual (19, 36, 40) or group CBT (34), and from workbook-based CCT (41) to modified PASAT protocols (35, 37, 38, 42).

These findings should be interpreted with caution. First, the CBIs category encompassed clinically and mechanistically distinct interventions, ranging from CBT-based protocols to neurocognitive training approaches. Although the subgroup analyses into CBT and CCT partially address this heterogeneity, they do not fully resolve it, and findings should be interpreted as reflecting a broad augmentation concept rather than a single standardized treatment model. Also, six of the included studies raised some concerns in the risk-of-bias assessment (19, 34–36, 38, 41), and one study was rated as high risk (39), which may affect the overall quality of the evidence. Additionally, the small overall sample size (321 patients across 10 studies) limits the statistical power of the pooled analyses, and the absence of follow-up data precludes assessment of the maintenance of treatment effects over time; most included studies did not report outcomes beyond the acute treatment period.

Although most studies reported MDD as the primary diagnosis, two studies included distinct depressive presentations, namely acute depressive disorder and late-life depression (35, 41). Additionally, one study reported MDD as a comorbidity of social anxiety disorder (36). Finally, prioritizing BDI and BDI-II when multiple depression scales were reported was not prespecified in the protocol and was decided *post hoc* based on data availability and consistency. A notable strength of this review is the low heterogeneity observed across all reported outcomes, which supports the consistency and robustness of the findings. To our knowledge, this is one of the first meta-analyses to examine tDCS combined with CBT-related therapies as a distinct subgroup.

## 5 Conclusion

These findings indicate that current sham-controlled evidence does not demonstrate a reliable incremental benefit of adjunctive tDCS over sham. When the analysis was restricted to CBT, results remained consistent, with no significant between-group differences. This review suggests that current evidence does not support a reliable additional effect of tDCS on depressive symptoms, response, remission, or quality of life beyond the underlying interventions.

Nevertheless, these findings should be interpreted with caution, given methodological variability and sample-related limitations. Future RCTs should use stronger methods, adequately powered samples, standardized protocols, and longer follow-up to clarify whether specific patient subgroups, treatment conditions, or stimulation parameters influence the role of tDCS as an adjunct to CBIs.

## Data Availability

The original contributions presented in the study are included in the article/**Supplementary Material**. Further inquiries can be directed to the corresponding author.
