# Supplementary material for: Transcranial direct current stimulation combined with cognitive-behavioral interventions for depressive disorders: a systematic review and meta-analysis of sham-controlled randomized trials
Source: Front Psychiatry. 2026 Aug 26;17:1899231. doi: 10.3389/fpsyt.2026.1899231 (PMC13555226; doi:10.3389/fpsyt.2026.1899231)
Supplement: Supplementary file 1 [file DataSheet1.pdf]

SUPPLEMENTARY MATERIAL

Transcranial Direct Current Stimulation Combined with Cognitive-Behavioral Interventions for Depressive Disorders: A Systematic Review and Meta-analysis of Sham-Controlled Randomized Trials.

Number of Figures: 5

Number of Tables: 5

SUPPLEMENTARY TABLES AND FIGURES:

Supplementary Table 1. Search strategy for all databases.

Supplementary Table 2. Included Studies.

Supplementary Table 3. Characteristics of included studies.

Supplementary Table 4. Prisma Checklist.

Supplementary Table 5. Evidence Certainty Assessment with GRADE.

Supplementary Figure 1. Sensitivity analysis including only studies that used the Beck Depression Inventory (BDI) as the depressive symptom outcome measure, assessing the outcome of reduction in depressive symptoms.

Supplementary Figure 2. Sensitivity analysis including only studies classified as having a low risk of bias according to RoB 2, assessing the outcome of reduction in depressive symptoms.

Supplementary Figure 3. Sensitivity analysis restricted to studies using clinician-rated scales, such as the MADRS and HAM-D, as the depressive symptom outcome measure, assessing the reduction in depressive symptoms.

Supplementary Figure 4. Sensitivity analysis comparing studies according to the timing of intervention delivery, with tDCS administered either concurrently with CBIs or separately, assessing the reduction in depressive symptoms

Supplementary Figure 5. Sensitivity analysis comparing studies according to tDCS session duration, assessing the reduction in depressive symptoms.

TABLES

Supplementary Table 1. Search strategy for all databases.

**Search Strategy**
(Depression OR Depressions OR "Major Depressive Disorder" OR MDD OR Depressive OR Depress*) AND ("Transcranial Direct Current Stimulation" OR tDCS OR "Direct Current Stimulation" OR "transcranial electrical stimulation" OR tES) AND ("Cognitive Behavioural Therapy" OR "Cognitive Behavioral Therapy" OR CBT OR "cognitive therapy" OR "behaviour therapy" OR "behavior therapy" OR "behavioural activation" OR "behavioral activation" OR BA OR "exposure therapy" OR "prolonged exposure" OR "problem-solving therapy" OR PST OR "rational emotive behaviour therapy" OR REBT OR "mindfulness-based cognitive therapy" OR MBCT OR "mindfulness-based stress reduction" OR MBSR OR mindfulness OR mindful* OR "acceptance and commitment therapy" OR ACT OR "dialectical behaviour therapy" OR "dialectical behavior therapy" OR DBT OR "compassion-focused therapy" OR CFT OR "metacognitive therapy" OR MCT OR "schema therapy" OR "self-system therapy" OR "interpersonal therapy" OR IPT OR "couples therapy" OR "marital therapy" OR "family therapy" OR "systemic therapy" OR "attachment-based therapy" OR "short-term psychodynamic therapy" OR "psychodynamic therapy" OR "supportive psychotherapy" OR "supportive therapy" OR counselling OR counseling OR "counselling therapy" OR "counseling therapy" OR "person-centred therapy" OR "humanistic therapy" OR "nondirective therapy" OR "psychoeducation" OR "social skills training" OR "relaxation training" OR "progressive muscle relaxation" OR "applied relaxation" OR biofeedback OR neurofeedback OR "behaviour modification" OR "behavior modification" OR psychotherapy OR "psychological therapy" OR "talk therapy") AND (Sham OR "Sham tDCS" OR placebo OR control OR "treatment as usual" OR "usual care") AND ("randomized controlled trial" OR "controlled clinical trial" OR randomized OR randomly OR trial OR "clinical trial" OR groups)

Supplementary Table 2. Included Studies.

Study	DOI
Aust 2022	10.1001/jamapsychiatry.2022.0696
Brunoni 2014	http://dx.doi.org/10.1016/j.jad.2014.03.026
Carvalho 2025	https://doi.org/10.3390/brainsci15050444
Ha 2024	https://doi.org/10.1016/j.brs.2024.02.009
Sararudi 2025	https://doi.org/10.1186/s12888-025-06866-5
Segrave 2014	http://dx.doi.org/10.1016/j.brs.2013.12.008
Sommer 2021	https://doi.org/10.1016/j.brs.2021.06.004
Vanderhasselt 2015	http://dx.doi.org/10.1016/j.pnpbp.2014.09.015
Nord 2019	https://doi.org/10.1038/s41386-019-0401-0
Welch 2017	10.1111/ner.12807

Supplementary Table 3. Characteristics of the Included Studies.

**Study**	**Mean Age (Years)**	**Stimulation Timing**	**Dropout/Completion**	**Response Definition**	**Remission Definition**
Aust 2022	42.7 ± 12.7 / 39.0 ± 14.8	Concurrent	5/48 active; 5/47 sham	≥50% MADRS reduction	MADRS ≤10
Brunoni 2014	46.1 ± 10.4 / 41.5 ± 10.6	Concurrent	High attrition by week 4; evenly distributed	Approx. 25% response	HDRS ≤7
Carvalho 2025	33.3 / 31.2	Sequential / booster-phase combined design	NR	≥12-point MADRS reduction	BDI ≤9
Ha 2024	76.0 ± 7.2 / 74.8 ± 4.0	Concurrent	1 active and 2 sham not analyzed	NR	NR
Nord 2019	35.6 ± 12.9 /	Sequential pre-	1/20 active; 5/19	≥50% HAM-D	HAM-D ≤7

	31.1 ± 8.2	CBT stimulation	sham	reduction	
Sararudi 2025	23.3 ± 6.5 / 26.0 ± 7.8	Sequential intensified stimulation before CBT	NR	NR	NR
Segrave 2014	42.6 ± 18.3 / 31.2 ± 5.7	Concurrent	1 posttreatment withdrawal	≥50% MADRS reduction	MADRS ≤10
Sommer 2021	34.6 ± 13.7 / 41.1 ± 16.2 / 37.7 ± 11.9	Concurrent	NR	NR	NR
Vanderhasselt 2015	46.3 ± 10.7 / 41.0 ± 11.5	Concurrent	4/37 total	NR	NR
Welch 2017	Wide range 32–67 years	Concurrent	4/14 total; mostly active-arm withdrawals	≥50% reduction descriptively reported	NR

Supplementary Table 4. PRISMA Checklist.

Section and Topic	Item #	Checklist item	Location where item is reported
**TITLE**			
Title	1	Identify the report as a systematic review.	Page 1
**ABSTRACT**			
Abstract	2	See the PRISMA 2020 for Abstracts checklist.	Page 4
**INTRODUCTION**			
Rationale	3	Describe the rationale for the review in the context of existing knowledge.	Pages 5 and 6
Objectives	4	Provide an explicit statement of the objective(s) or question(s) the review addresses.	Pages 5 and 6
**METHODS**			
Eligibility criteria	5	Specify the inclusion and exclusion criteria for the review and how studies were grouped for the syntheses.	Page 6
Information sources	6	Specify all databases, registers, websites, organisations, reference lists and other sources searched or consulted to identify studies. Specify the date when each source was last searched or consulted.	Page 6

Section and Topic	Item #	Checklist item	Location where item is reported
Search strategy	7	Present the full search strategies for all databases, registers and websites, including any filters and limits used.	Supplementary Material
Selection process	8	Specify the methods used to decide whether a study met the inclusion criteria of the review, including how many reviewers screened each record and each report retrieved, whether they worked independently, and if applicable, details of automation tools used in the process.	Pages 6 and 7
Data collection process	9	Specify the methods used to collect data from reports, including how many reviewers collected data from each report, whether they worked independently, any processes for obtaining or confirming data from study investigators, and if applicable, details of automation tools used in the process.	Page 6
Data items	10a	List and define all outcomes for which data were sought. Specify whether all results that were compatible with each outcome domain in each study were sought (e.g. for all measures, time points, analyses), and if not, the methods used to decide which results to collect.	Pages 6 and 7
	10b	List and define all other variables for which data were sought (e.g. participant and intervention characteristics, funding sources). Describe any assumptions made about any missing or unclear information.	
Study risk of bias assessment	11	Specify the methods used to assess risk of bias in the included studies, including details of the tool(s) used, how many reviewers assessed each study and whether they worked independently, and if applicable, details of automation tools used in the process.	Page 7
Effect measures	12	Specify for each outcome the effect measure(s) (e.g. risk ratio, mean difference) used in the synthesis or presentation of results.	Page 7
Synthesis methods	13a	Describe the processes used to decide which studies were eligible for each synthesis (e.g. tabulating the study intervention characteristics and comparing against the planned groups for each synthesis (item #5)).	Page 7
	13b	Describe any methods required to prepare the data for presentation or synthesis, such as handling of missing summary statistics, or data conversions.	Pages 6 and 7
	13c	Describe any methods used to tabulate or visually display results of individual studies and syntheses.	Pages 8 to 12 and Supplementary Materials.
	13d	Describe any methods used to synthesize results and provide a rationale for the choice(s). If meta-analysis was performed, describe the model(s), method(s) to identify the presence and extent of statistical heterogeneity, and software package(s) used.	Pages 6 and 7

Section and Topic	Item #	Checklist item	Location where item is reported
	13e	Describe any methods used to explore possible causes of heterogeneity among study results (e.g. subgroup analysis, meta-regression).	Page 7 and 12
	13f	Describe any sensitivity analyses conducted to assess robustness of the synthesized results.	Pages 10,11 and Supplementary Materials.
Reporting bias assessment	14	Describe any methods used to assess risk of bias due to missing results in a synthesis (arising from reporting biases).	Page 7
Certainty assessment	15	Describe any methods used to assess certainty (or confidence) in the body of evidence for an outcome.	Page 7 and Supplementary Material
**RESULTS**			
Study selection	16a	Describe the results of the search and selection process, from the number of records identified in the search to the number of studies included in the review, ideally using a flow diagram.	Pages 7 and 8
	16b	Cite studies that might appear to meet the inclusion criteria, but which were excluded, and explain why they were excluded.	Page 7 and Supplementary Materials
Study characteristics	17	Cite each included study and present its characteristics.	Pages 8 and 9
Risk of bias in studies	18	Present assessments of risk of bias for each included study.	Pages 10, 11 and 12
Results of individual studies	19	For all outcomes, present, for each study: (a) summary statistics for each group (where appropriate) and (b) an effect estimate and its precision (e.g. confidence/credible interval), ideally using structured tables or plots.	Pages 9, 10 and 11
Results of syntheses	20a	For each synthesis, briefly summarise the characteristics and risk of bias among contributing studies.	Pages 10,11 and 12
	20b	Present results of all statistical syntheses conducted. If meta-analysis was done, present for each the summary estimate and its precision (e.g. confidence/credible interval) and measures of statistical heterogeneity. If comparing groups, describe the direction of the effect.	Pages 9, 10, 11 and Supplementary Materials
	20c	Present results of all investigations of possible causes of heterogeneity among study results.	Pages 9, 10, 11, 12 and Supplementary Materials

Section and Topic	Item #	Checklist item	Location where item is reported
	20d	Present results of all sensitivity analyses conducted to assess the robustness of the synthesized results.	Page 11 and Supplementary Materials
Reporting biases	21	Present assessments of risk of bias due to missing results (arising from reporting biases) for each synthesis assessed.	Pages 10,11 and 12
Certainty of evidence	22	Present assessments of certainty (or confidence) in the body of evidence for each outcome assessed.	Supplementary Materials
**DISCUSSION**			
Discussion	23a	Provide a general interpretation of the results in the context of other evidence.	Pages 12, 13 and 14
	23b	Discuss any limitations of the evidence included in the review.	Pages 13 and 14
	23c	Discuss any limitations of the review processes used.	Page 14
	23d	Discuss implications of the results for practice, policy, and future research.	Page 14
**OTHER INFORMATION**			
Registration and protocol	24a	Provide registration information for the review, including register name and registration number, or state that the review was not registered.	Page 6
	24b	Indicate where the review protocol can be accessed, or state that a protocol was not prepared.	Page 6
	24c	Describe and explain any amendments to information provided at registration or in the protocol.	Page 14
Support	25	Describe sources of financial or non-financial support for the review, and the role of the funders or sponsors in the review.	Page 15
Competing interests	26	Declare any competing interests of review authors.	Page 11
Availability of data, code and other materials	27	Report which of the following are publicly available and where they can be found: template data collection forms; data extracted from included studies; data used for all analyses; analytic code; any other materials used in the review.	Page 15

Supplementary Table 5. Evidence Certainty Assessment with GRADE.

TDCS + CBIs compared to Sham tDCS + CBIs for Depressive Disorders

Patient or population: Major Depressive Disorder

Setting:

Intervention: tDCS + Cognitive-Behavioral Therapies

Comparison: Sham tDCS + Cognitive-Behavioral Therapies

Outcomes	№ of participants (studies) Follow-up	Certainty of the evidence (GRADE)	Relative effect (95% CI)	Anticipated absolute effects	
				Risk with Sham tDCS + Cognitive-Behavioral Therapies	Risk difference with tDCS + Cognitive-Behavioral Therapies
Patients Response Rate assessed with: Number of Events	203 (6 RCTs)	⊕○○○ Very low[a,b,c]	**RR 1.08** (0.67 to 1.74)	302 per 1.000	**24 more per 1.000** (from 100 fewer to 224 more)
Patients Remission Rate assessed with: Number of Events	203 (6 RCTs)	⊕○○○ Very low[a,b,c]	**RR 1.33** (0.74 to 2.38)	208 per 1.000	**69 more per 1.000** (from 54 fewer to 288 more)
Reduction on Depressive Symptoms assessed with: Standardized Mean Difference	339 (9 RCTs)	⊕⊕○○ Low[a,b]	-	-	SMD **0.03 SD lower** (0.3 lower to 0.23 higher)
Improvement in Quality of Life assessed with: Standardized Mean Difference	120 (3 RCTs)	⊕○○○ Very low[b,d,e]	-	-	SMD **0.06 SD higher** (0.61 lower to 0.73 higher)

***The risk in the intervention group** (and its 95% confidence interval) is based on the assumed risk in the comparison group and the **relative effect** of the intervention (and its 95% CI).

CI: confidence interval; **RR:** risk ratio; **SMD:** standardised mean difference

---

GRADE Working Group grades of evidence

High certainty: we are very confident that the true effect lies close to that of the estimate of the effect.

Moderate certainty: we are moderately confident in the effect estimate: the true effect is likely to be close to the estimate of the effect, but there is a possibility that it is substantially different.

Low certainty: our confidence in the effect estimate is limited: the true effect may be substantially different from the estimate of the effect.

Very low certainty: we have very little confidence in the effect estimate: the true effect is likely to be substantially different from the estimate of effect.

Explanations

a. Most of included studies were rated as having some concerns, and one study was rated as having a high risk of bias.

b. Intervention parameters and neuromodulation settings were highly heterogeneous across studies. In addition, comorbidities were frequent.

c. Only six studies were included, and the confidence interval was relatively wide.

d. All included studies were rated as having some concerns.

e. Only three studies were included.

FIGURES

Supplementary Figure 1. Sensitivity analysis including only studies that used the Beck Depression Inventory (BDI) as the depressive symptom outcome measure, assessing the outcome of reduction in depressive symptoms.

Insert Supplementary Figure 1

Supplementary Figure 2. Sensitivity analysis including only studies classified as having a low risk of bias according to RoB 2, assessing the outcome of reduction in depressive symptoms.

Insert Supplementary Figure 2

Supplementary Figure 3. Sensitivity analysis restricted to studies using clinician-rated scales, such as the MADRS and HAM-D, as the depressive symptom outcome measure, assessing the reduction in depressive symptoms.

Insert Supplementary Figure 3

Supplementary Figure 4. Sensitivity analysis comparing studies according to the timing of intervention delivery, with tDCS administered either concurrently with CBIs or separately, assessing the reduction in depressive symptoms.

Insert Supplementary Figure 4

Supplementary Figure 5. Sensitivity analysis comparing studies according to tDCS session duration, assessing the reduction in depressive symptoms.

Insert Supplementary Figure 5
